# Systematic review on infection and disease caused by *Thelazia callipaeda* in Europe: 2001–2020

**DOI:** 10.1051/parasite/2020048

**Published:** 2020-09-29

**Authors:** Beatriz do Vale, Ana Patrícia Lopes, Maria da Conceição Fontes, Mário Silvestre, Luís Cardoso, Ana Cláudia Coelho

**Affiliations:** 1 Department of Veterinary Sciences, School of Agrarian and Veterinary Sciences (ECAV), University of Trás-os-Montes e Alto Douro (UTAD) 5000-801 Vila Real Portugal; 2 Animal and Veterinary Research Centre, UTAD 5000-801 Vila Real Portugal; 3 Department of Zootechnics, ECAV, UTAD 5000-801 Vila Real Portugal

**Keywords:** Companion animals, Europe, Systematic review, *Thelazia callipaeda*, Thelaziosis, Wildlife

## Abstract

Cases of thelaziosis by *Thelazia callipaeda* have been increasing considerably in Europe throughout the 21st century, with recent emphasis on Eastern Europe. A systematic review was conducted using defined search terms across three major databases and, additionally, with the examination of the references of the 56 articles selected. Available information about epidemiological and clinical features of all cases of thelaziosis by *T*. *callipaeda* in companion animals, wildlife and humans was extracted, evaluated and subjected to qualitative and quantitative analysis. In all cross-sectional studies about dogs, cats and red foxes, males were more frequently infected than females (dogs: *p* = 0.0365; cats: *p* = 0.0164; red foxes: *p* = 0.0082). Adult dogs seem to be more prone to infection (*p* < 0.0001), as well as large-sized dogs (*p* < 0.0001), and companion animals that live exclusively outdoors (*p* < 0.0001). Dogs and red foxes involved in these cross-sectional studies harboured significantly more female than male nematodes (*p* < 0.0001). Thelaziosis by *T*. *callipaeda* is far from controlled in Europe. Only through updated epidemiological data, knowledge improvement and awareness can correct diagnosis and appropriate treatment and prevention be ensured to tackle this zoonosis.

## Abstract (fr)

Les cas de thélaziose par *Thelazia callipaeda* ont considérablement augmenté en Europe tout au long du 21e siècle, l’accent étant récemment mis sur l’Europe de l’Est. Une revue systématique a été menée en utilisant des termes de recherche définis dans trois grandes bases de données, et aussi en examinant les références des 56 articles sélectionnés. Les informations disponibles sur les caractéristiques épidémiologiques et cliniques de tous les cas de thélaziose par *T. callipaeda* chez les animaux de compagnie, la faune et les humains ont été extraites, évaluées et soumises à une analyse qualitative et quantitative. Dans toutes les études transversales sur les chiens, les chats et les renards roux, les mâles étaient plus fréquemment infectés que les femelles (chiens : *p* = 0,0365; chats : *p* = 0,0164; renards roux : *p* = 0,0082). Les chiens adultes semblent être plus sujets aux infections (*p* < 0,0001), ainsi que les chiens de grande taille (*p* < 0,0001) et les animaux de compagnie vivant exclusivement à l’extérieur (*p* < 0,0001). Les chiens et les renards roux impliqués dans ces études transversales abritaient significativement plus de nématodes femelles que de mâles (*p* < 0,0001). La thélaziose par *T. callipaeda* est loin d’être contrôlée en Europe. Ce n’est que grâce à des données épidémiologiques mises à jour, à l’amélioration des connaissances et à la sensibilisation que l’on pourra corriger le diagnostic et assurer un traitement et une prévention appropriés pour lutter contre cette zoonose.

## Introduction

Members of the genus *Thelazia* (Spirurida, Thelaziidae) dwell the orbital cavity and associated tissues of several mammals and birds [1], and at least 16 species have been identified in different hosts [49, 75]. However, only *Thelazia callipaeda* Railliet & Henry, 1910, *Thelazia californiensis* Price, 1930 and *Thelazia gulosa* Railliet & Henry, 1910 have been found to affect humans [2–4, 19].

*Thelazia callipaeda* is also known as “the oriental eyeworm” because of its distribution throughout the former Soviet Union and East Asia [1, 59, 70]. Nevertheless, at the end of the 20th century, autochthonous cases were reported in Italy [50, 52] and, since then, *T*. *callipaeda* has increasingly been reported in some European countries both in animals and humans [75]. In Europe, the intermediate host of this eyeworm is the male drosophilid fruit fly *Phortica variegata* Fallén, 1823 (Drosophilidae, Steganinae), which feeds on lachrymal secretions of mammals [53, 55, 58, 60].

There are reported cases of thelaziosis in companion animals (dogs and cats), lagomorphs such as hares (*Lepus europaeus*) and wild rabbits (*Oryctolagus cuniculus*), but also in wild carnivores, especially red foxes (*Vulpes vulpes*), which appear to play an important role in the introduction and geographical dispersion of this eyeworm in non-endemic European regions [15, 23, 32, 44, 51, 57].

The importance of studying and investigating thelaziosis lies in the fact that *T*. *callipaeda* has a broad spectrum of hosts and the number of infected hosts, including humans, in Europe has been increasing since the beginning of the 21st century. Taking into account that thelaziosis is an expanding disease, it becomes necessary to identify the characteristics of all the cases reported, in order to profile this zoonosis. Therefore, the objective of the present study was to carry out a qualitative and quantitative analysis based on a systematic review of the scientific literature. Consequently, it is expected that this compilation of all reported cases will be useful in future investigations on understanding the evolution of thelaziosis by *T*. *callipaeda*.

## Materials and methods

### Study design

The present study consisted of a systematic review of the literature in order to answer the following research question: “What are the epidemiological and clinical features as well as the prevalence of thelaziosis by *T*. *callipaeda* reported in companion animals, wildlife and humans in Europe in the 21st century?” This study was conducted based on the methodological recommendations of the Preferred Reporting Items for Systematic Reviews and Meta-Analyses (PRISMA) [46].

### Article eligibility

Articles published in indexed journals cited in PubMed, ScienceDirect and Web of Science were considered eligible if they consisted of case reports or cross-sectional studies describing clinical and epidemiological features (including species, prevalence, gender, age, country, geographical area, affected eye, and ocular signs). There were other restrictions regarding the eligibility criteria. Only studies published between 1 January 2001 and 10 July 2020, and written in the languages of Western Europe (comprising English, French, German, Italian, Portuguese and Spanish) were included.

The types of publications included original articles, short communications and case reports that addressed issues within the following criteria: (i) information on the clinical presentation of thelaziosis by *T. callipaeda* in companion animals, wild animals and humans; and (ii) prevalence of the disease in canine, feline and red fox populations.

Reviews of the literature, research notes, editorials, experimental essays, textbook chapters, posters, abstracts, articles with no primary data and dissertations along with unpublished studies and data were excluded.

### Information sources and search strategies

The process of identifying articles in indexed journals was developed using the PubMed, ScienceDirect and Web of Science databases. The combination of search terms in English applied included: {Europe AND (*Thelazia callipaeda* OR thelaziosis OR thelaziasis OR thelazi*)}. To prevent missing data, references of retrieved publications were also checked in order to identify additional papers. The searches were conducted between March and July 2020.

### Selection of studies and data extraction

After a comprehensive systematic searching, a bibliographic manager tool (Zotero version 5.0.88) [65] was used to exclude duplicate records. Then, two independent reviewers selected articles based on their titles and abstracts, followed by a full reading of the text when the title or abstract met the inclusion criteria or could not be rejected with certainty. Any disagreements or divergences were resolved by discussion and consensus.

Two researchers extracted the required data and added the information on an electronic spreadsheet, dividing them into three groups: (i) companion animals (dog, cat, and domestic rabbit), (ii) wildlife, and (iii) humans. The qualitative data about companion animals comprised references (country, authors and year of publication), case report data (host species, breed, age, gender, lifestyle, and affected eye), number of infected animals, number of adult nematodes and ocular signs (Table 1). With regard to wildlife, information was extracted about the authors, year of publication, country, case report (species, gender and affected eye), number of infected animals, number of adult nematodes and ocular signs (Table 2). For cases of human thelaziosis, references (country, region, data on authors and year of publication), gender, age, affected eye, number of infected hosts, number of adult nematodes and ocular symptoms were collected (Table 3). In turn, the quantitative data extracted from the second group of articles (cross-sectional studies) comprised the references (authors and year of publication), country where the study was conducted, species (dogs, cats and red foxes) and their identification, sample size, number of positive animals, and prevalence (%).

**Table 1:** Features of the studies included in the qualitative analysis regarding *T*. *callipaeda* infection in companion animals in Europe.

References		Sample		Clinical aspects	
Country	Authors/year	Species	No. of affected animals	Affected eye	Ocular signs
Austria	Hodžić et al. [29]	Cat	1	Right	Conjunctival edema
					Conjunctival hyperemia
					Serous discharge
Belgium	Caron et al. [9]	Dog^a^	2	Left	Conjunctivitis
					Follicular hyperplasia of the 3rd eyelid
					Purulent discharge
Bosnia and Herzegovina	Hodžić et al. [28]	Cat	1	Left	Conjunctival hyperemia
		Dog	4	Unilateral or both	Conjunctivitis
Bulgaria	Colella et al. [10]	Dog	9	NA	Conjunctivitis
					Epiphora
Croatia	Hodžić et al. [28]	Dog	2	NA	Conjunctival hyperemia
					Conjunctivitis
Czech Republic	Jirků et al. [34]	Dog	1	Right	Purulent discharge
France	Dorchies et al. [14]	Cat	1	Left	Chemosis
					Conjunctivitis
					Wound on the 3rd eyelid
		Dog	4	NA	Conjunctivitis
France^b^	Ruytoor et al. [66]	Cat	2	NA	NA
		Dog	115	NA	NA
Germany	Hermosilla et al. [27]	Dog^c^	1	Right	Conjunctivitis
					Purulent discharge
Germany	Schottstedt [68]	Dog	1	Right	Blepharospasm
					Conjunctivitis
					Keratitis
					Purulent discharge
Germany	Magnis et al. [38]	Dog	1	Right	Blepharospasm
					Epiphora
					Lacrimation
Germany	Silva et al. [71]	Cat^d^	1	NA	Epiphora
					Mucoid discharge
		Dog^e^	2	Right	Conjunctival hyperemia
					Epiphora
					Follicular conjunctivitis
					Mucoid or mucopurulent discharge
Greece	Diakou et al. [12]	Dog	1	Both	Conjunctival edema
					Conjunctivitis
					Epiphora
					Keratitis
					Mucoid discharge
Greece	Papadopoulos et al. [60]	Cat	3	NA	Conjunctivitis
		Dog	46	NA	Anterior uveitis
					Blepharitis
					Conjunctivitis
					Corneal abrasions
					Indolent corneal ulcer
					Mucoid or mucopurulent discharge
		Rabbit^f^	1	NA	Conjunctivitis
					Mucoid discharge
Hungary	Colella et al. [10]	Dog	1	NA	Conjunctivitis
					Epiphora
Hungary	Farkas et al. [18]	Cat	1	Right	Conjunctival hyperemia
					Conjunctivitis
					Purulent discharge
		Dog	10	Both (6)	Conjunctival hyperemia
				Left (2)	Conjunctivitis
				Right (2)	Purulent discharge
Moldova	Dumitrache et al. [16]	Dog	1	Right	Conjunctivitis
					Epiphora
					Periocular alopecia
					Periocular erosions
Portugal	Pimenta et al. [63]	Dog	1	Left	Blepharospasm
					Epiphora
Portugal	Rodrigues et al. [64]	Cat	1	Left	Conjunctival edema
					Conjunctivitis
					Infra-orbital abscess
Portugal	Vieira et al. [76]	Dog	9	NA	Conjunctivitis
					Ocular discharge
					Ocular pruritus
Portugal	Soares et al. [72]	Cat	1	Right	Blepharospasm
					Conjunctival edema
					Photophobia
					Purulent discharge
Romania	Mihalca et al. [43]	Dog	1	Right	Conjunctivitis
					Epiphora
					Proliferative lesions inferior conjunctival sac
Romania	Ioniţă et al. [33]	Dog	2	Right	Conjunctivitis
					Epiphora
					Mild lesions inferior conjunctival sac
Romania	Tudor et al. [74]	Dog	2	Right	Conjunctivitis
					Mucopurulent discharge
				Both	Blepharospasm
Romania	Dumitrache et al. [15]	Cat	1	Left	Blepharospasm
					Conjunctival edema
					Mucopurulent discharge
					Periocular alopecia
					Periocular erosions
					Photophobia
		Dog	5	Both (2)	Blepharitis
				Right (1)	Conjunctivitis
				Left (2)	Epiphora
Serbia	Gajić et al. [21]	Cat	2	Left	Conjunctival edema
					Conjunctival hyperemia
		Dog	4	Both (2)	Conjunctivitis
				Left (2)	Ocular discharge
Serbia	Tasić-Otašević et al. [73]	Dog	2	Unilateral	Chemosis
					Conjunctival hyperemia
Slovakia	Čabanová et al. [8]	Dog	4	Both (1)	Conjunctivitis
				Left (1)	Epiphora
				Right (2)	
Spain	Marino et al. [42]	Dog	1001^g^	NA	Chemosis
					Conjunctivitis
					Corneal ulceration
					Follicular conjunctivitis
					Follicular hyperplasia of the 3rd eyelid Purulent discharge
Switzerland	Malacrida et al. [40]	Cat	5	NA	Conjunctivitis
					Epiphora
		Dog	106^h^	NA	Conjunctivitis
					Epiphora
					Keratitis
Turkey	Eser et al. [17]	Dog	1	Left	Conjunctivitis
					Purulent discharge
United Kingdom	Graham-Brown et al. [24]	Dog^i^	3	Both (1)	Conjunctival hyperemia
				Left (1)	Conjunctivitis
					Corneal ulceration
				Right (1)	Increased blink rate
					Serous discharge
United Kingdom	Hammond [26]	Dog^j^	1	Right	Conjunctivitis

NA: Not available

a The infections had been acquired in south western France and southern Italy.

b Most cases (104/117) were diagnosed in the Dordogne department. Furthermore, most of the infected animals in the other regions had spent time in Dordogne a few months before clinical signs developed.

c The dog had visited several parts of Italy for a few weeks before clinical signs developed.

d The cat was imported from Southern Spain to West Germany (Freinshein).

e One dog was imported from Kenya and the other was in close contact with several dogs from different countries at racing events, which took place in the Netherlands and Austria.

f *Oryctolagus cuniculus*.

g *n* = 11 autochthonous cases of dogs in 6 municipalities of Andorra.

h Out of 95 *Thelazia*-positive dogs with known data about travel history, 40 had previously been to Italy (one year before diagnosis).

i One dog had been imported to the UK from Hateg, Hunedoara county (western Romania) 6 weeks previously. The other dog had returned from travel to the Lombardy region (northern Italy). The third dog had spent 1 month at the Saint Avit Loisirs campsite, Dordogne department (France).

j The dog presented unilateral conjunctivitis 1 month after returning from a 2-week stay in the Dordogne department (France).

**Table 2:** Features of the studies included in the qualitative analysis regarding *T*. *callipaeda* infection in wildlife in Europe.

References		Sample		Clinical aspects	
Country	Authors/year	Species	No. of affected animals	Affected eye	Ocular signs
Italy	Otranto et al. [56]	Wolf^a^	3	Both (2)	NA
				Right (1)	
Italy	Otranto et al. [57]	Wolf	1	Left	NA
		Beech marten^b^	3	Both	
		Brown hare^c^	3	Both	
		Wild cat^d^	3	Both	
Portugal	Sargo et al. [67]	Red fox^e^	1	Both	Conjunctivitis
		Red fox	2	NA	
Portugal	Gama et al. [23]	Wild rabbit^f^	2	Both	None
Portugal	Seixas et al. [69]	Beech marten	1	Left	None
Romania	Mihalca et al. [44]	Golden jackal^g^	1	Both	NA
		Wolf	1	NA	
		Wildcat	1	Left	
Romania	Ionică et al. [32]	Beech marten	1	Right	NA
		European badger^h^	1	Both	NA
Serbia	Pavlović et al. [62]	Red fox	3	Left	NA
Serbia	Gajić et al. [22]	Wolf	8	Both	NA
Spain	Calero-Bernal et al. [7]	Red fox	2	Both Right	NA
Spain	Nájera et al. [48]	Wolf	1	Both	NA

NA: not available.

a Wolf – *Canis lupus.*

b Beech marten – *Martes foina.*

c Brown hare – *Lepus europaeus.*

d Wild cat – *Felis silvestris.*

e Red fox – *Vulpes vulpes*.

f Wild rabbit – *Oryctolagus cuniculus.*

g Golden jackal – *Canis aureus.*

h European badger – *Meles meles*.

**Table 3:** Features of the studies included in the qualitative analysis regarding *T*. *callipaeda* infection in humans.

References			Clinical aspects	
Country	Region	Authors/year	Affected eye	Ocular symptoms
Croatia	Slavonski Brod	Paradžik et al.^a^ [61]	Left	Conjunctival and ciliary infection
				Conjunctival hyperemia
				Corneal abscess
				Itching
				Lacrimation
				Ocular pain and discomfort
France	Nice	Otranto and Dutto [50]	Both	Exsudative conjunctivitis
				Foreign body sensation
				Lacrimation
Germany	Essen	Dolff et al. [13]	Left	Foreign body sensation
				Lacrimation
				Redness
Italy	Roja Valley^b^	Otranto and Dutto [50]	Right	Exsudative conjunctivitis
				Foreign body sensation
				Lacrimation
Italy	Canelli	Otranto and Dutto [50]	Right	Exsudative conjunctivitis
				Foreign body sensation
				Lacrimation
Italy	Cuneo	Otranto and Dutto [50]	Right	Exsudative conjunctivitis
				Foreign body sensation
				Lacrimation
Serbia	Medveđa	Tasić-Otašević et al. [73]	Left	Itching
				Ocular pain and discomfort
				Redness
Spain	Coria	Fuentes et al. [20]	Left	Exsudative conjunctivitis
				Foreign body sensation
				Lacrimation
Spain	El Bierzo	López-Medrano et al. [37]	Right	Conjunctival hyperemia
				Foreign body sensation
				Lacrimation
Spain	Ourense	Deltell et al. [11]	NA	Conjunctival hyperemia
				Tarsal reaction
Spain	Ourense	Deltell et al. [11]	NA	Conjunctival hyperemia
				Tarsal reaction

NA: not available.

a Croatian patient living in a rural household in close contact with a dog.

b This patient from Liguria had gone trekking in the woods in Tenda (Piedmont region, Italy), approximately 3 weeks before the onset of clinical signs.

### Data analysis

The data collected were analysed using IBM SPSS Statistics 26 statistical software [30]. A Chi-square test was used to test associations between the parameters. Statistical significance was considered if *p* value was < 0.05.

## Results

**Figure 1:**
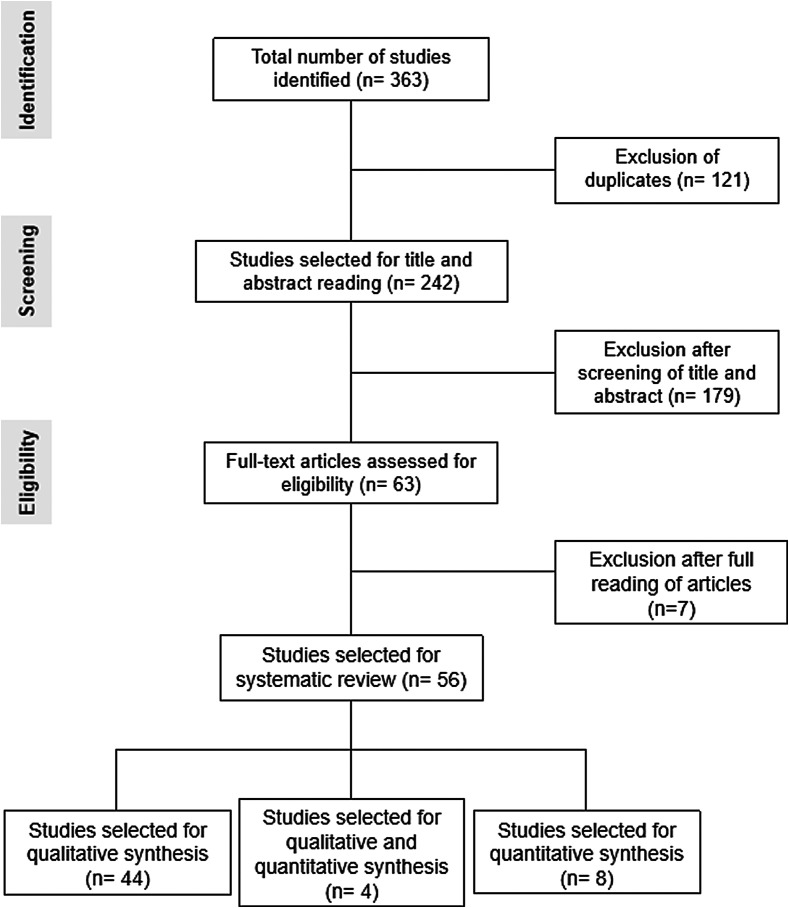
Flow chart of the search, selection and inclusion process for studies in the systematic review based on PRISMA guidelines.

The identification and study selection are represented in Figure 1. Articles that did not meet the inclusion criteria, as well as duplicates and incomplete articles or studies not available online or in other sources, were excluded. Thus, out of the 363 studies searched, 56 met the eligibility criteria and were divided according to the subject they addressed: epidemiological and clinical characteristics of thelaziosis (*n* = 44), which were included in the qualitative analysis; studies with relevant data to both qualitative and quantitative analysis (*n* = 4); or the prevalence of thelaziosis by *T. callipaeda* in dogs, cats and red foxes (*n* = 8), which were considered to be cross-sectional (prevalence) studies with data for quantitative analysis.

### Qualitative analysis of the epidemiological and clinical aspects of *T*. *callipaeda* infection in companion animals

The 31 studies included in the qualitative analysis of thelaziosis in companion animals had been conducted in 19 different countries (Austria, Belgium, Bosnia and Herzegovina, Bulgaria, Croatia, Czech Republic, France, Germany, Greece, Hungary, Moldova, Portugal, Romania, Serbia, Slovakia, Spain, Switzerland, Turkey and the United Kingdom) (Table 1). They reported occurrence of *T*. *callipaeda* infection in dogs (*n* = 1343), cats (*n* = 20), and a rabbit (*n* = 1), in a total of 1364 animals (Table 1).

The studies reported the gender of 206 dogs, of which 76 were female (36.9%) and 130 were male (63.1%); 16 cats, of which six were female (37.5%) and 10 were male (62.5%); and the rabbit, which was male.

It was possible to obtain the age of 161 dogs (161/1343, 12.0%), one of which was less than 1 year old (1/161, 0.6%), 113 dogs were 1–8 years old (113/161, 70.2%) and 47 dogs were more than 8 years old (47/161, 29.2%); of the 16 cats whose age was reported (16/20, 80.0%), one cat was more than 8 years old (1/16, 6.3%) and the remaining 15 cats were 1–8 years old (15/16, 93.8%). The rabbit was more than 8 years old.

Of the 58 dogs whose breed was reported, 38 dogs were purebred (38/58; 65.5%) and 20 were crossbred (20/58; 34.5%). The main represented purebred was Labrador Retriever (*n* = 5; 13.5%), followed by Collie, German Shepherd and Golden Retriever (*n* = 4 each; 10.5% each), Portuguese Podengo and Transmontano Mastiff (*n* = 2 each; 5.3% each), Beagle, Borzoi, Dalmatian, Brittany, Greek Hound, Hungarian Vizla, Jack Russel Terrier, Large Münsterländer, Patterdale Terrier, Rottweiler, Samoyed, Scottish Terrier, Sharplanina, Siberian Husky, Spitz, West White Highland Terrier and Wirehaired Slovakian Pointer (*n* = 1 each; 2.6% each). In cats, the main represented breed was European shorthair (11/15; 73.3%), followed by Greek shorthair (3/15; 20%) and Brown Tabby European (1/15; 6.7%).

It was possible to collect data on body size of 134 dogs (134/1343; 10.0%), most of which fitted in the large category (*n* = 100; 74.6%), followed by medium (*n* = 20; 14.9%) and small (*n* = 14; 10.4%).

The lifestyle of 179 dogs was reported (179/1343, 13.3%), 50 of which lived strictly outdoors (50/179, 27.9%) and 129 lived between inside and outside the house (129/179, 72.1%); regarding the ten cats with information about their lifestyle (10/20, 50.0%), five lived between indoors and outdoors (5/10, 50.0%) and five lived exclusively outdoors (5/10, 50.0%). The domestic rabbit had an exclusively outdoor lifestyle.

In dogs (*n* = 45; 3.4%), the most affected eye was the right one (*n* = 19; 42.2%) followed by both eyes (*n* = 14; 31.1%) and the left eye (*n* = 12; 26.7%). In turn, the most affected eye in cats was the left one (*n* = 6; 66.7%) followed by the right eye (*n* = 3; 33.3%) (Table 1).

The number of specimens of *T*. *callipaeda* was counted in 65 dogs (65/1343, 4.8%) and 14 cats (14/20, 70.0%), making a total of 79 animals (79/1363, 5.8%). In both host species, the number of female nematodes (dogs: *n* = 600; cats: *n* = 43) was higher than the number of male nematodes (dogs: *n* = 265; cat: *n* = 18). The gender of parasitic specimens collected from both domestic species was determined for 89.2% (931/1044).

The 28 articles reporting canine thelaziosis referred to clinical manifestations such as conjunctivitis (*n* = 21), epiphora (*n* = 10), purulent discharge (*n* = 6), blepharospasm (*n* = 4), conjunctival hyperemia (*n* = 5), keratitis (*n* = 3), blepharitis (*n* = 2), chemosis (*n* = 2), corneal ulceration (*n* = 2), follicular conjunctivitis (*n* = 3), follicular hyperplasia of the 3rd eyelid, (*n* = 2), ocular discharge (*n* = 3), anterior uveitis, conjunctival edema, corneal abrasions, increased blink rate, indolent corneal ulcer, lacrimation, mild and proliferative lesions of the inferior conjunctival sac, mucoid discharge, mucoid or mucopurulent discharge, ocular pruritus, periocular alopecia and erosions and serous discharge (*n* = 1 in each study). The 12 studies reporting feline thelaziosis described ocular signs, including conjunctival oedema (*n* = 5), conjunctivitis (*n* = 4), conjunctival hyperaemia (*n* = 3), blepharospasm (*n* = 2), epiphora (*n* = 2), photophobia (*n* = 2), chemosis, infra-orbital abscess, mucopurulent or purulent or serous discharge, periocular alopecia and erosions and wound on the 3rd eyelid (*n* = 1 each) (Table 1). The domestic rabbit had conjunctivitis and mucoid ocular discharge (Table 1).

### Qualitative analysis of the epidemiological and clinical aspects of *T*. *callipaeda* infection in wildlife

The 11 studies included in the qualitative analysis of thelaziosis in wildlife were conducted in five different countries (i.e. Italy, Portugal, Romania, Serbia and Spain) (Table 2). They reported occurrences of thelaziosis by *T*. *callipaeda* in beech martens (*Martes foina*) (*n* = 5), brown hares (*Lepus europaeus*) (*n* = 3), European badger (*Meles meles*) (*n* = 1), golden jackal (*Canis aureus*) (*n* = 1), red foxes (*Vulpes vulpes*) (*n* = 8), wild rabbits (*Oryctolagus cuniculus*) (*n* = 2), wildcats (*Felis silvestris*) (*n* = 4) and gray wolves (*Canis lupus*) (*n* = 14), in a total of 38 animals (Table 2).

The studies reported the gender of all animals, except five red foxes. Out of the five beech martens, three were female (3/5, 60%) and two were male (2/5, 40%); the three brown hares were female, the European badger was female and the golden jackal was male; of the three red foxes whose gender was reported, one was female (1/3, 33.3%) and two were male (2/3, 66.7%); one wild rabbit was female and the other one was male; out of the four wildcats, one was female (1/4; 25%) and three were male (3/4; 75%); out of the 14 wolves, four were female (4/14; 28.6%) and the remaining 10 were male (10/14; 71.4%).

In all host species, with the exception of red foxes, both eyes were the most affected (beech marten: 3/5, 60%; brown hare: 3/3, 100%; European badger: 1/1, 100%; golden jackal: 1/1, 100%; wild rabbit: 2/2, 100%; wildcat: 3/4, 75%; wolf: 11/13, 84.6%), followed by the left eye (beech marten: 1/5, 20%; red fox: 3/6, 50%; wildcat: 1/4, 25%; wolf: 1/13, 7.7%) and the right eye (beech marten: 1/5, 20%; red fox: 1/6, 16.7%; wolf: 1/13, 7.7%). There were no reports on this topic for two red foxes and one wolf (Table 2).

A total of 615 specimens of *T*. *callipaeda* were collected from the wildlife mentioned in the studies, and the gender of the nematodes was identified in 610 specimens (610/ 615; 99.2%). The number of female nematodes (beech marten: 10/17, 58.8%; brown hare: 14/17, 82.4%; golden jackal: 21/29, 72.4%; red fox: 35/54, 64.8%; wild rabbit: 5/6, 83.3%; wolf: 313/441, 71.0%) was higher than the number of male nematodes (beech marten: 7/17, 41.2%; brown hare: 3/17, 17.7%; golden jackal: 8/29, 27.6%; red fox: 14/54, 25.9%; wild rabbit: 1/6, 16.7%; wolf: 128/441, 29.0%) in all species, with the exception of wildcats, in which the number of female and male nematodes was equal (9 female and 9 male) and the European badger, in which more males than females were counted (female: 10/33, 30.3%; male: 23/33, 69.7%).

Out of the 11 studies reporting thelaziosis in wildlife, only one study described ocular signs (conjunctivitis) on red foxes (Table 2).

### Qualitative analysis of the epidemiological and clinical aspects of *T*. *callipaeda* infection in humans

The seven studies included in the qualitative synthesis of *T*. *callipaeda* infection in humans reported 11 cases in six different countries: Croatia (*n* = 1), France (*n* = 1), Germany (*n* = 1), Italy (*n* = 3), Serbia (*n* = 1) and Spain (*n* = 4).

Out of the 11 cases, nine were male hosts (81.8%) and two were female hosts (18.2%), with ages between 15 and 35 years (*n* = 3), 35 and 55 years (*n* = 4), 55 and 75 years (*n* = 3) and over 75 years (*n* = 1).

Out of the nine cases whose affected eyes were reported, four cases occurred in the right eye (4/9, 44.4%), four in the left eye (4/9, 44.4%), and one case in both eyes (1/9, 11.1%).

A total of 17 specimens of *T*. *callipaeda* were collected from the human cases mentioned in five out of the seven studies. However, the gender of nematodes was only identified in eight specimens, in which four were female (4/8, 50.0%) and four were male (4/8, 50.0%).

All studies reported ocular signs in the affected humans, including lacrimation (*n* = 8), foreign body sensation (*n* = 7), exudative conjunctivitis (*n* = 5), conjunctival hyperaemia (*n* = 4), itching (*n* = 2), ocular pain and discomfort (*n* = 2), tarsal reaction (*n* = 2), conjunctival and ciliary infection (*n* = 1), corneal abscess (*n* = 1), and redness (*n* = 2) (Table 3).

### Quantitative analysis of the epidemiological and clinical aspects of *T*. *callipaeda* infection in dogs, cats and red foxes

Regarding canine thelaziosis, seven studies were carried out in Italy, Portugal, Serbia, Spain and Switzerland (Table 4). Of a total of 659 positive dogs, the gender of 366 animals is known (females: 163, 44.5%; and males: 203, 55.5%).

**Table 4:** Quantitative analysis regarding the main features of the studies about *T*. *callipaeda* infection in dogs, cats and red foxes in Europe.

Species	References	Country	Sample	Positive	Prevalence (%)
Dog	Otranto et al. [52]	Italy	91	21	23.1
	Malacrida et al. [40]	Switzerland	529	28	5.29
	Miró et al. [45]	Spain	456	182	39.9
	Hadži-Milić et al. [25]	Serbia	501	178	35.5
	Maia et al. [39]	Portugal	586	22	3.75
	Marino et al. [41]	Spain	287	115	40.1
	Marino et al. [42]	Spain	137	84	61.3
	Marino et al. [42]	Spain	97	29	29.9
Cat	Motta et al. [47]	Switzerland	2171	17	0.8
	Maia et al. [39]	Portugal	27	4	23.5
Red fox	Otranto et al. [57]	Italy	75	37	49.3
	Malacrida et al. [40]	Switzerland	126	7	5.6
	Hodžić et al. [28]	Bosnia and Herzegovina	184	51	27.7
	Čabanová et al. [6]	Slovakia	523	7	1.3
	Ionică et al. [31]	Romania	514	151	29.4

To date, there are only two cross-sectional studies referring to thelaziosis by *T*. *callipaeda* in cats. These studies were conducted in Portugal and Switzerland and their prevalences were 23.5% and 0.8%, respectively (Table 4). They reported the occurrence of *T. callipaeda* infection in 21 cats (females: 5, 23.8%; males: 16, 76.2%).

Regarding wildlife, five cross-sectional studies have already been carried out in order to determine the prevalence of *T*. *callipaeda* infection in red foxes. These studies took place in Bosnia and Herzegovina, Italy, Romania, Slovakia and Switzerland (Table 4). Most red foxes came from legal hunts during rabies-monitoring programmes, so their eye examination occurred *post-mortem* at necropsy. Two hundred and fifty-three positive red foxes were reported, of which 105 were female (105/252, 41.7%) and 147 were male (147/252, 58.3%) (the gender of one red fox was unknown).

*Thelazia*-positive dogs from quantitative analysis (cross-sectional studies) indicated a significantly higher occurrence in male dogs (*p* = 0.0365). The same scenario was observed in cats (*p* = 0.0164) and red foxes (*p* = 0.0082).

It was possible to obtain the age of 86 dogs (86/659, 13.1%), having significant differences between young, adult and senior dogs (*p* < 0.0001), since 16 of which were less than 1 year old (16/86, 18.6%), 55 dogs were 1–8 years old (55/86, 64.0%), and 15 dogs were more than 8 years old (15/86, 17.4%); of the 17 cats whose age was reported, one cat was less than 1 year old (1/17, 5.9%), 14 cats were 1–8 years old (14/17, 82.4%), and two cats were more than 8 years old (2/17, 11.8%).

In dogs, there were statistically significant differences regarding the breed (*p* = 0.0095), with crossbred dogs (30/43, 69.8%) being more infected than purebred dogs (13/43, 30.2%). The breed of 17 cats was known, all of which were crossbred.

Regarding the body size effect, there were statistically significant differences between positive dogs (*p* < 0.0001). Small-sized dogs (47/307, 15.3%) have been found to be less infected than medium-sized dogs (122/307, 39.7%), followed by large-sized dogs (138/307, 45.0%).

The lifestyle of 515 dogs was reported, and those that lived strictly outdoors (482/515, 93.6%) were found to be significantly more infected than those that lived inside and outside the house (33/515, 6.4%) (*p* < 0.0001). Regarding the 17 cats with information about their lifestyle, all of them lived between indoors and outdoors.

Taking into account the information available for the “infected eye”, it was found that in 33 dogs both eyes were infected (33/178, 18.5%), while 145 dogs harboured unilateral infection (145/178, 81.5%). Including the 33 dogs that harboured bilateral infection, the left eye (108/211, 51.2%) was more frequently infected than the right eye (103/211, 48.8%), but without statistical significance (*p* = 0.7307). The same scenario was observed in cats (left eye: 11/18, 61.1%; right eye: 7/18, 38.9%; *p* = 0.3458) and in red foxes (left eye: 202/403, 50.1%; right eye: 201/403, 49.9%; *p* = 0.9603).

The number of female nematodes (dogs: 676/940, 71.9%; cats: 11/17, 64.7%; red foxes: 2428/3534, 68.7%) was higher than the number of male nematodes (dogs: 264/940, 28.1%; cats: 6/17, 35.3%; red foxes: 1106/3534, 31.3%) (Table 5). Dogs and red foxes involved in these cross-sectional studies harboured significantly more female than male nematodes (*p* < 0.0001), whereas in cats this difference was not statistically significant (*p* = 0.2253). It is important to note that the gender of all parasitic specimens collected from red foxes was not determined (total: 391/3925, 10.0%).

**Table 5:** Gender identification and intensity of *T. callipaeda* infection in the eyes of dogs, cats and red foxes from the cross-sectional studies.

Species	References	Positive animals	*Thelazia callipaeda*	Intensity of infection					
			Total	Female	Male	Sex ratio (F:M)	Min.	Max.	mean ± SD
Dog	Otranto et al. [52]	21	NA	NA	NA	NA	NA	NA	NA
	Malacrida et al. [40]	28	NA	NA	NA	NA	1	23	NA
	Miró et al. [45]	182	762	548	214	2.6:1	1	28	4.2 ± 4.73
	Hadži-Milić et al. [25]	178	NA	NA	NA	NA	1	78	NA
	Maia et al. [39]	22	178	128	50	2.6:1	1	35	8.08 ± 9.49
	Marino et al. [41]	115	NA	NA	NA	NA	NA	NA	NA
	Marino et al. [41]	113	NA	NA	NA	NA	NA	NA	9.2 ± 16.7
	Total	659	940	676	264				
Cat	Motta et al. [47]	17	NA	NA	NA	NA	1	10	2.8
	Maia et al. [39]	4	17	11	6	1.8:1	1	14	4.3 ± 6.5
	Total	21	17	11	6				
Red fox	Malacrida et al [40]	7	27	NA	NA	NA	1	10	NA
	Otranto et al. [57]	37	139	97	42	2.3:1	1	13	3.8 ± 2
	Hodžić et al. [28]	51	364	NA	NA	NA	1	50	8.08 ± 9.41
	Čabanová et al. [6]	7	45	31	14	2.2:1	2	12	6.4 ± 4.4
	Ionică et al. [31]	151	3350	2300	1050	2.2:1	1	192	23.2
	Total	253	3925	2428	1106				

NA: not available, SD: standard deviation.

All studies reported a sex ratio in favour of females. Also, the intensity of infection with adult forms of *T*. *callipaeda* per study shows high variability, as indicated in Table 5.

## Discussion

The occurrence of *T*. *callipaeda* infection was significantly higher in male dogs, cats and red foxes from cross-sectional studies, and an equal outcome was observed in the qualitative analysis. In addition, there is no evidence of a host sex predisposition, but there are contradictory results concerning this, as has been shown in previous reports [40, 45, 52]. In relation to cats, it is known that in the samples from the study by Motta et al. [47] all the hosts were male and had outdoor access, which could be risk factors for *T*. *callipaeda* infection, possibly due to their territorial and hunting behaviour, but also because of a wider roaming area. Perhaps a similar situation regarding the possible male predisposition in red foxes may be assumed, given that the quantitative analysis has shown that male foxes were significantly more affected than female foxes.

It was observed that the age group corresponding to the adult phase (1–8 years old) was the most affected, having significant differences between the three age groups of dogs. This may be due to the fact that adults potentially had more access to outdoor spaces than juvenile and elderly dogs, a circumstance that predisposed them to contact with *P*. *variegata*. This result is in line with that observed in cats [47], as well as in the qualitative analysis of the present study. Nevertheless, some studies have not reported significant differences in infected animals when ages were compared [45, 52].

In relation to the possible breed effect, on the quantitative analysis, there were statistical significant differences regarding the breed, with crossbred dogs being more frequently infected than purebred dogs. Nonetheless, given that the sample consisted of only 43 dogs, it is not possible to confirm that crossbred dogs are more susceptible to infection than purebred dogs. In addition, there is no article that points out in that direction and whose qualitative analysis shows a different perspective. Likewise, in previous reports, no effect of breed was detected [45, 52]. Nevertheless, when it comes to aptitude/management, there seems to be a greater tendency for crossbred or purebred dogs, whose aptitude is shepherd or hunting, being infected by *T*. *callipaeda*, since they have a higher possibility for physical contact with the vector while outside in the forest environment [5, 8, 39].

Another feature that appears to play an important role in infection with *Thelazia* is body size. Small-sized dogs have been found to be less frequently infected than medium-sized dogs, followed by large-sized dogs. This can be explained by the fact that most large-sized dogs are usually housed outdoors, a circumstance that increases their exposure to the intermediate hosts [40, 45, 75]. Additionally, it is accepted that a larger body surface also favours physical contact with *P*. *variegata* [40]. In contrast, the lower prevalence in small-sized dogs and in cats may be due to their small body mass index, which apparently makes them less attractive to the intermediate host. Moreover, cats eliminate eye discharges through their intensive cleaning habits, therefore losing their decoy to flies [40, 47, 52]. In addition, feline infection seems to be underestimated because of difficulties experienced by veterinarians inspecting cats’ eyes [15, 47].

As already reported, lifestyle seems to be an important feature in the occurrence of *Thelazia* infection. In fact, animals that frequently (or exclusively) live outdoors are more highly exposed to *P*. *variegata* flies. Similarly, places where there is physical contact between animals, which are potential hosts, seem to attract the intermediate host [39, 45].

In the three species submitted to a quantitative analysis, it was found that the left eye was the most frequently affected. However, these results come from few sources and represent a small sample [6, 25, 28, 31, 40, 47, 57]. Consequently, it is not possible to compare or infer about eye predisposition. Moreover, to the best of our knowledge, there is no scientific article that has identified the existence of a pattern with respect to the most affected eye by *T*. *callipaeda.*

Thelaziosis has already been described, based on cases reports and prevalence studies, in several European countries, having as the common denominator the zoophilic fruit fly *P*. *variegata*, which is the intermediate host of *T*. *callipaeda*. All cases reported have occurred in regions characterised by similar altitude (800–1000 a.s.l) and climate and habitat conditions: continental Mediterranean climate, and cultivated areas and deciduous woods, which fall within the geoclimatic model for the distribution of *P*. *variegata* [54].

A higher prevalence of *T*. *callipaeda* infection, especially in dogs and red foxes, as described in Spain [41, 45] or Italy [57], suggests a stable endemic condition [42] (Table 4). A lower prevalence of *T*. *callipaeda*, such as in Portugal [39] or Slovakia [6], could be associated with the recent emergence of the infection at the local level [42] (Table 4). However, this outcome is expected to be underdiagnosed and/or underreported. The number of companion animals and wildlife (not only red foxes) positive to *T*. *callipaeda* might be considerably higher in the mentioned countries, but also in other European countries that have not yet performed prevalence studies. It is important to raise awareness about the need to perform these studies in order to understand the European reality of this zoonosis.

Canine thelaziosis was firstly reported in southern Europe, in Italy, and then in western Europe and the Balkan area. Subsequently, more and more cases appeared in eastern Europe, and it is evident that this zoonosis is already established in Central Europe [5, 10, 29, 34, 35, 38, 51]. The spread of thelaziosis in endemic regions in Europe, but also to previously non-endemic areas, has been linked to the migration of infected wild animals, especially red foxes as well as wolves, jackals and other wildlife. This demonstrates the role of free-ranging wild carnivores as reservoirs of *T*. *callipaeda* and highlights the importance of the sylvatic cycle, especially in rural areas, where transmission to humans and domestic animals is facilitated [6, 28, 34, 44, 57]. However, the role of pets, especially dogs, in spreading the disease, should not be neglected [35, 36]. The movement of pets when traveling with their owners within the European Union, but also the adoption and import of dogs from shelters from endemic regions are a crucial driver of *Thelazia*, but also for other pathogenic agents and their vectors [16, 34]. Undoubtedly, these risk factors highlight the importance of preventive programmes [35, 36] and surveillance polices to restrict cross-border spread of the nematode [34, 75].

## Conclusions

In this work, isolated cases of thelaziosis were summarized and an in-depth analysis of all cross-sectional studies was conducted. The reduced number of prevalence studies and the small sample per study were the main disadvantages found, as this made it difficult or even impossible to infer or determine the situation for certain features. However, this work shows the expansion potential of *T*. *callipaeda* and the urgent need for additional large-scale studies in order to provide information on the current situation in the European Union. Given the scarcity of papers on human health, the need to stress the importance of the One Health approach is sustained. Only through updated epidemiological data, knowledge improvement, and awareness can correct diagnosis and appropriate treatment and prevention of thelaziosis be ensured.
